# Noncultured epidermal cell suspension for the treatment of recalcitrant segmental vitiligo in a solid-organ transplant recipient^☆^^☆☆^

**DOI:** 10.1016/j.abd.2019.02.014

**Published:** 2020-02-12

**Authors:** Gerson Dellatorre, Caio César Silva de Castro

**Affiliations:** a Department of Dermatology, Hospital Santa Casa de Misericórdia de Curitiba, Curitiba, PR, Brazil; b Escola de Medicina, Pontifícia Universidade Católica do Paraná, Curitiba, PR, Brazil

Dear Editor,

Vitiligo is an acquired disease that affects 0.5% of the Brazilian population characterized by loss of melanocytes from the epidermis.^1^

Segmental vitiligo (SV) presents as macules and patches commonly distributed in a unisegmental form which do not cross body midline.^1^ It is characterized by the rapid onset of the disease and may present a limited response to clinical treatments, especially if initiated late.^2^ Besides, concerns about ultraviolet-related carcinogenesis in Solid-organ transplant (SOT) recipients can limit this treatment modality in this population.^3^

We present a case of SV in SOT recipient treated by noncultured epidermal cell suspension (NCES) without complimentary phototherapy.

A 31-year-old male patient presented with a history of achromic macules and patches on his left arm distributed in a segmental form. The lesions began five years ago when the patient was under pegylated interferon alfa-2a treatment due to an IgA nephropathy associated with hepatitis B. After his diagnosis of VS he treated the lesions with topical betamethasone dipropionate cream 0.05% and tacrolimus ointment 0.1% for four months without improvement. The patient also underwent 85 treatments of narrowband UVB therapy, with only 25% improvement. His nephropathy worsened, and the patient started hemodialysis followed by renal transplantation.

Skin lesions at the time of consultation were distributed in a blaschkoid fashion and were clinically compatible with SV stable for the last four years.

Due to the risk of carcinogenesis, we contraindicated more phototherapic treatments and planned the NCES procedure. First, we obtained a thin partial-thickness skin graft with 8 cm^2^ under local anesthesia from his left medial thigh with the assistance of a shaving blade. Then, the graft was incubated at 37 °C in a trypsin 0.25% with Ethylenediaminetetraacetic Acid (EDTA) solution for fifty minutes. After that, we used a fine forceps to separate the epidermis from the dermis, allowing detachment of basal epidermal cells in normal saline. The solution was transferred to a test tube, and we obtained a cell pellet after five minutes centrifugation at 1500 rpm.

The recipient site was delimited (Fig. 1) and prepared by superficial dermabrasion under local anesthesia (lidocaine 1% without epinephrine). The cell pellet was resuspended in 1.5 mL of normal saline and transferred to the recipient site. A collagen sheet, petrolatum gauze, regular gauze, and transparent adhesive film were positioned over the area and dressings were kept in place for seven days.

**Figure 1:**
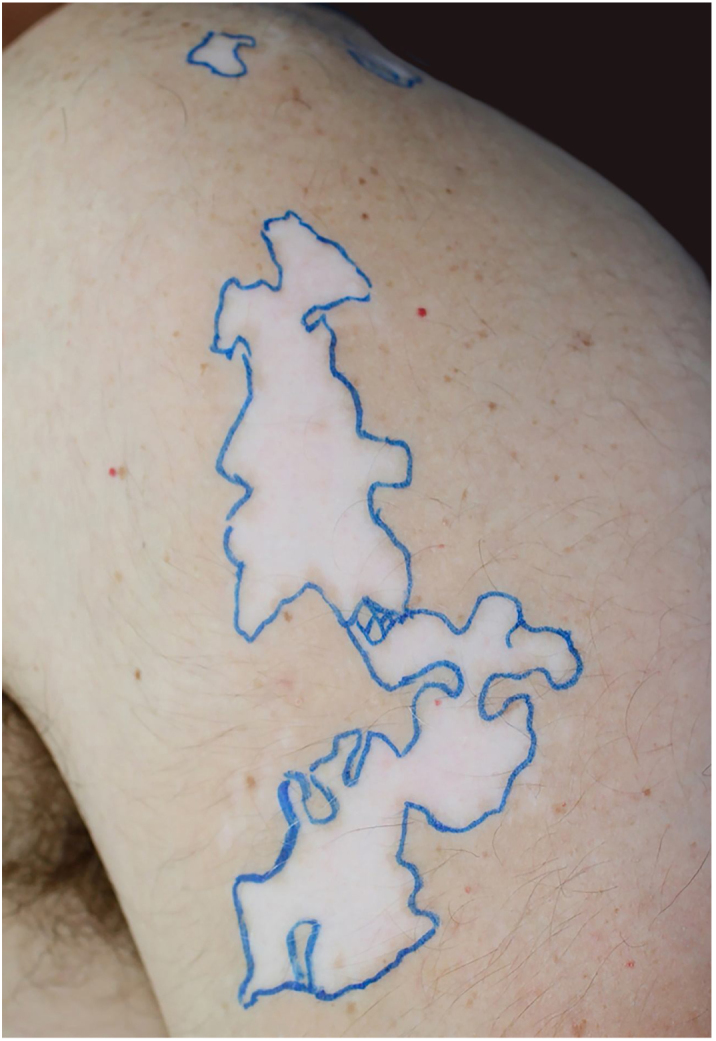
Achromic patches with blaschkoid distribution demarcated on the left arm (24 cm^2^).

At the time of the procedure, the patient was on the use of everolimus 1 mg/day, tacrolimus 3 mg/day and prednisone 10 mg/day since the last year. Due to his immunosuppression, we prescribed oral antibiotic therapy (cefadroxil 500 mg b.i.d.) for seven days. The patient did not receive postoperative phototherapy.

After three months a diffuse repigmentation on 95% of the recipient site started to become visible, becoming more evident after six months (Figure 2, Figure 3).

**Figure 2:**
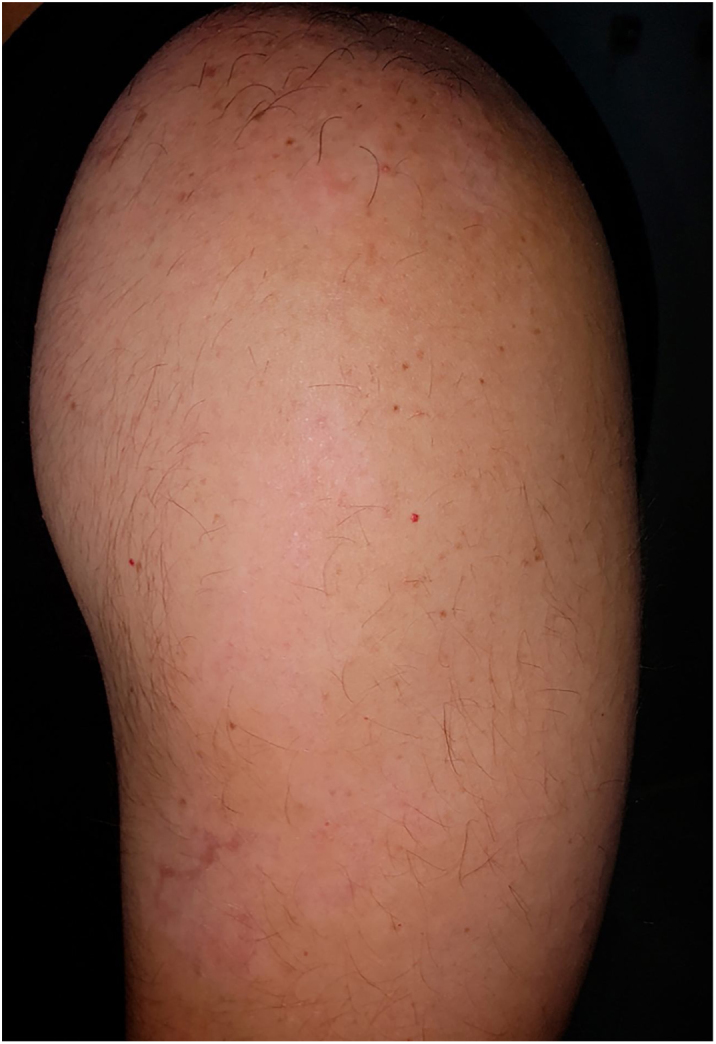
Repigmentation on 95% of recipient site after six months with slight hypochromia.

**Figure 3:**
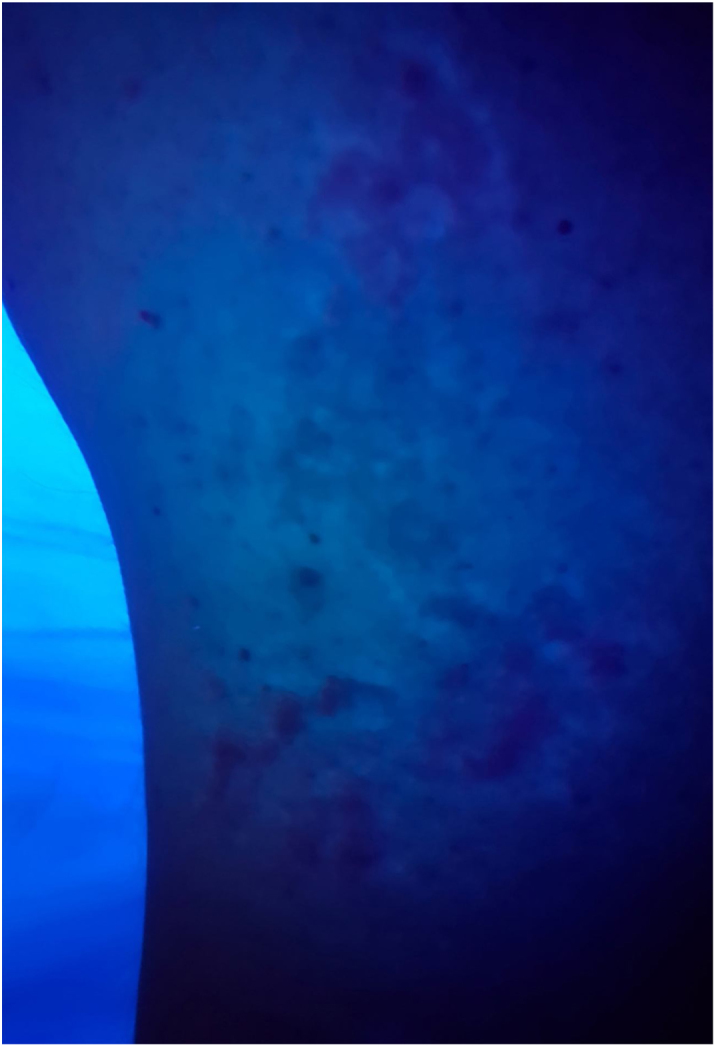
Wood's lamp examination shows diffuse repigmentation pattern.

Main therapeutic approaches for SV include corticosteroids, topical immunomodulators and phototherapy. Although phototherapy is considered a first-line treatment in vitiligo,^1^ the response of SV to modalities such as UVBnb is limited. A Korean study showed repigmentation rates varying from 26.3% to 50% in SV patients treated with UVBnb.^2^

Besides, although NBuvb studies have not found a significant association between treatment modality and keratinocyte carcinomas or melanomas in immunocompetent patients,^4^ special considerations must be done regarding this treatment in SOT population. Compared with the general population, SOT recipients are between 65 and 250 times more likely to develop skin spindle cell carcinomas.^5^

Our patient presented with vitiligo lesions which were stable for five years. The disease stability is the main criteria in the indication of surgical treatment of vitiligo, once its presence is directly related to better outcomes.^1^ In theory, due to chronic use of immunosuppressants drugs, SOT population tends to present a more stable disease, being good candidates to the surgical procedure.

Although there is evidence supporting the use of pre and postsurgical phototherapy to improve outcomes in the surgical management of vitiligo, we can consider NCES without phototherapy as monotherapy for situations such as SOT patients, in which UV exposure is contraindicated.

## Financial support

None declared.

## Authors’ contributions

Gerson Dellatorre: Approval of the final version of the manuscript; conception and planning of the study; elaboration and writing of the manuscript; obtaining, analysis, and interpretation of the data; effective participation in research orientation; intellectual participation in the propaedeutic and/or therapeutic conduct of the studied cases; critical review of the literature; critical review of the manuscript.

Caio César Silva de Castro: Approval of the final version of the manuscript; conception and planning of the study; effective participation in research orientation; intellectual participation in the propaedeutic and/or therapeutic conduct of the studied cases; critical review of the manuscript.

## Conflicts of interest

None declared.
